# Follistatin like-1 aggravates silica-induced mouse lung injury

**DOI:** 10.1038/s41598-017-00478-0

**Published:** 2017-03-24

**Authors:** Yinshan Fang, Si Zhang, Xiaohe Li, Fangxin Jiang, Qiao Ye, Wen Ning

**Affiliations:** 1 State Key Laboratory of Medicinal Chemical Biology, College of Life Sciences, Nankai University, Tianjin, China; 2 Department of Occupational Diseases and Toxicology, Beijing Chao-Yang Hospital, Capital Medical University, Beijing, China

**Keywords:** Respiration, Respiratory tract diseases, Experimental models of disease

## Abstract

Occupational inhalation of dust, such as crystalline silica, for prolonged periods in the workplace leads to fibrotic lung diseases worldwide. The mechanisms underlying the diseases are unknown, so that no effective treatment exists for these conditions. We found elevated levels of follistatin like 1 (FSTL1) in serum from patients with silicosis and in lungs from silica-induced mouse model. The induced Fstl1 regulated inflammation response via activation of nod-like receptor family, pyrin domain containing 3v (NLRP3) inflammasome-mediated IL-1β production from macrophages. Meanwhile, Fstl1 promoted fibrosis via positive regulation of TGF-β1 signaling. Haploinsufficiency of *Fstl1* or blockage of FSTL1 with a neutralizing antibody was protective from silica-induced lung injury in mice *in vivo*. Our data suggest that Fstl1 plays an important role in lung fibrosis, and may serve as a novel therapeutic target for treatment of silicosis.

## Introduction

The lung is an inner organ connecting directly to the outside environment through breath and its structure and function are consistently affected by various airborne factors, including silica^1, 2^. Long-term occupational or environmental exposure to free crystalline silica particles causes silicosis^3, 4^, a significant occupational lung disease worldwide. Although prevention efforts have been made, the disorder occurs everywhere, most common among workers in developing countries but frequently even in developed countries. China has the most number of patients with silicosis in the world^3^, with 6000 new cases and more than 24,000 deaths reported annually. Silicosis and its increasing mortality have made silica exposure a high-priority public health concern in China, as well as in countries worldwide^5^.

Silicosis has been studied extensively. Its pathogenesis involves an early inflammatory response, followed by a fibrotic phase in which the extracellular matrix (ECM) is deposited and lung parenchyma is remodeled^6^. Inflammation is a hallmark of exposure to silica, during which, alveolar macrophages and their produced IL-1β have been suggested to play a crucial role. It is also known that many fibrogenic cytokines, such as TGF-β1^7^, have been demonstrated to be involved in the development of silicosis. Other molecular mediators, such as integrin^8^, CTGF^9^, amplify the fibrosis by regulating TGF-β1 responsiveness. However, the exact molecular mechanisms underlying remain poorly understood. Additional innovative studies are vital to diagnose these pneumoconioses in the early stages of development, and to develop treatment strategies.

Follistatin-like 1 (*Fstl1*), initially identified as a TGF-β1-inducible gene, encodes a secreted extracellular glycoprotein belonging to the secreted proteins rich in cysteine (SPARC) family^10^, whose amino acid sequence contains a follistatin-like domain and is highly conserved among species^11–13^. The growing literatures have identified Fstl1 as a putative tumor suppressor in lung and ovarian cancer cells^14, 15^, a dual-functional immunomodulator in human rheumatoid arthritis and other autoimmune diseases^16–19^, an Akt protein kinase-regulated cardioprotective factor in the heart^20^, and a BMP4 antagonist in embryonic development^21, 22^. However, its functions and mechanisms have not been fully elucidated. Fstl1 is found to be expressed in the pulmonary system and is crucial for lung development and homeostasis^23^. We have previously shown that Fstl1 is a profibrotic factor that is up-regulated in lungs of patients with IPF and bleomycin-injured mouse model^24^. We have demonstrated that the ectopic Fstl1 promotes the accumulation of myofibroblasts and subsequent fibrosis via balancing TGF-β1 and BMP4 signaling^24^. However, the role of Fstl1 in other types of lung fibrosis and their matched mouse models, such as silicosis and its related silica-induced mouse model, has not been investigated.

In this study, we confirm the profibrotic function of Fstl1 using a silica model of lung fibrosis and peripheral blood samples from patients with silicosis. We find that Fstl1 can increase NLRP3 inflammasome-mediated IL-1β secretion from macrophages. We further provide evidence that targeting FSTL1 with a neutralizing antibody may offer a potential therapeutic approach for patients with silicosis.

## Results

### Up-regulation of Fstl1 expression in silica-injured mice and patients with silicosis

**Figure 1:**
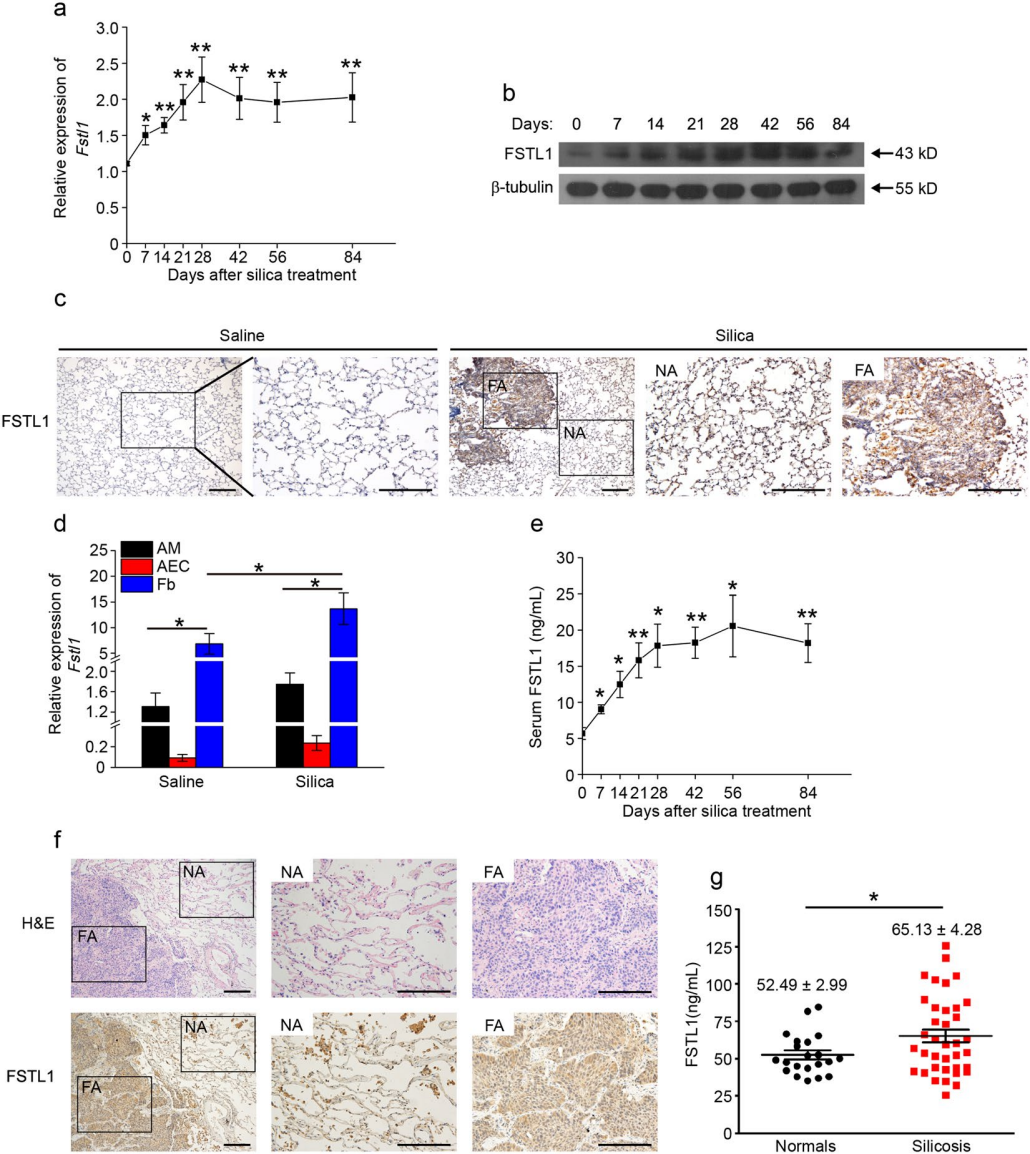
Up-regulation of Fstl1 in silica-injured mice and patients with silicosis. (**a**) *Fstl1* mRNA expression in lung tissues of C57BL/6J mice at the different time-point after silica injury was determined by qRT-PCR analysis (n = 6 per group; *P < 0.05; **P < 0.01 by one-way ANOVA followed by Student’s *t* test). (**b**) FSTL1 protein in lung tissues of C57BL/6J mice at the different time-point after silica injury was determined by western blot analysis. β-tubulin was used as a loading control. (**c**) Immunohistochemistry (IHC) of FSTL1 in lung sections of C57BL/6J mice 21 days after saline or silica injury. Representative images of the staining are shown. NA stands for normal area, FA stands for fibrotic area; both are shown at higher magnification. (n = 6 per group; scale bars, 200 μm). (**d**) *Fstl1* mRNA expression in primary alveolar macrophages (AMs), alveolar epithelial cells (AECs) and fibroblasts (Fb) at day 21 after saline or silica injury was determined by qRT-PCR analysis (n = 6 per group; *P < 0.05 by one-way ANOVA followed by Student’s *t* test). (**e**) FSTL1 protein in serum of C57BL/6J mice at the different time-point after silica injury was determined by ELISA analysis (n = 6 per group; *P < 0.05; **P < 0.01 by one-way ANOVA followed by Student’s *t* test). (**f**) Representative images of lung fibrotic area of patient with silicosis with H&E staining and immunohistochemical staining for FSTL1 protein (NA stands for normal area, FA stands for fibrotic area; Scale bars, 200 μm). (**g**) FSTL1 levels in serum of patients with silicosis and normal control individuals were determined by ELISA (*P < 0.05 by one-way ANOVA followed by Student’s *t* test).

Given the increased Fstl1 expression is observed in bleomycin model of lung fibrosis^24^, it is of interest to determine whether the expression of Fstl1 is altered in silica model of fibrosis. C57BL/6 J mice were intratracheally administrated with silica and Fstl1 expression was examined over time after injury. As shown in Fig. 1a and b, silica injury stimulated Fstl1 mRNA and protein expression in whole lung tissue in a time-dependent manner, maximal between day 21 - day 28, then stable till day 84. Immunohistochemical (IHC) examination of lung sections displayed considerably increased FSTL1 staining in active fibrotic areas (Fig. 1c). In addition, we found that the increased Fstl1 production was mainly from mesenchymal cells, as indicated by the significantly higher mRNA level of *Fstl1* in newly isolated lung fibroblasts than that of epithelial cells or alveolar macrophages (Fig. 1d). Considering that FSTL1 is a secreted glycoprotein, we further collected mouse peripheral blood and measured circulating FSTL1 levels with ELISA analysis. In agreement with the increased Fstl1 expression in lung tissue, elevated levels of circulating FSTL1 were detected in serum from silica-injured mice compared to those from control untreated mice (Fig. 1e).

To provide clinical evidence for the increase Fstl1 level in samples from experimental silica-induced fibrosis, we determined whether expression and circulating level of FSTL1 were altered in patients with silicosis. The enhanced expression of FSTL1 in active fibrotic area of biopsy with silicosis was confirmed by IHC staining (Fig. 1f). Circulating FSTL1 levels were quantified in serum from 37 patients with silicosis and 21 healthy controls. Circulating FSTL1 levels in serum from patients with silicosis were markedly higher than that in healthy individuals (Fig. 1g). Our data confirm that Fstl1 is up-regulated in response to silica injury and suggested that Fstl1 may play a role in the pathogenesis of silica-induced lung fibrosis.

### *Fstl1* deficiency attenuates silica-induced lung fibrosis

**Figure 2:**
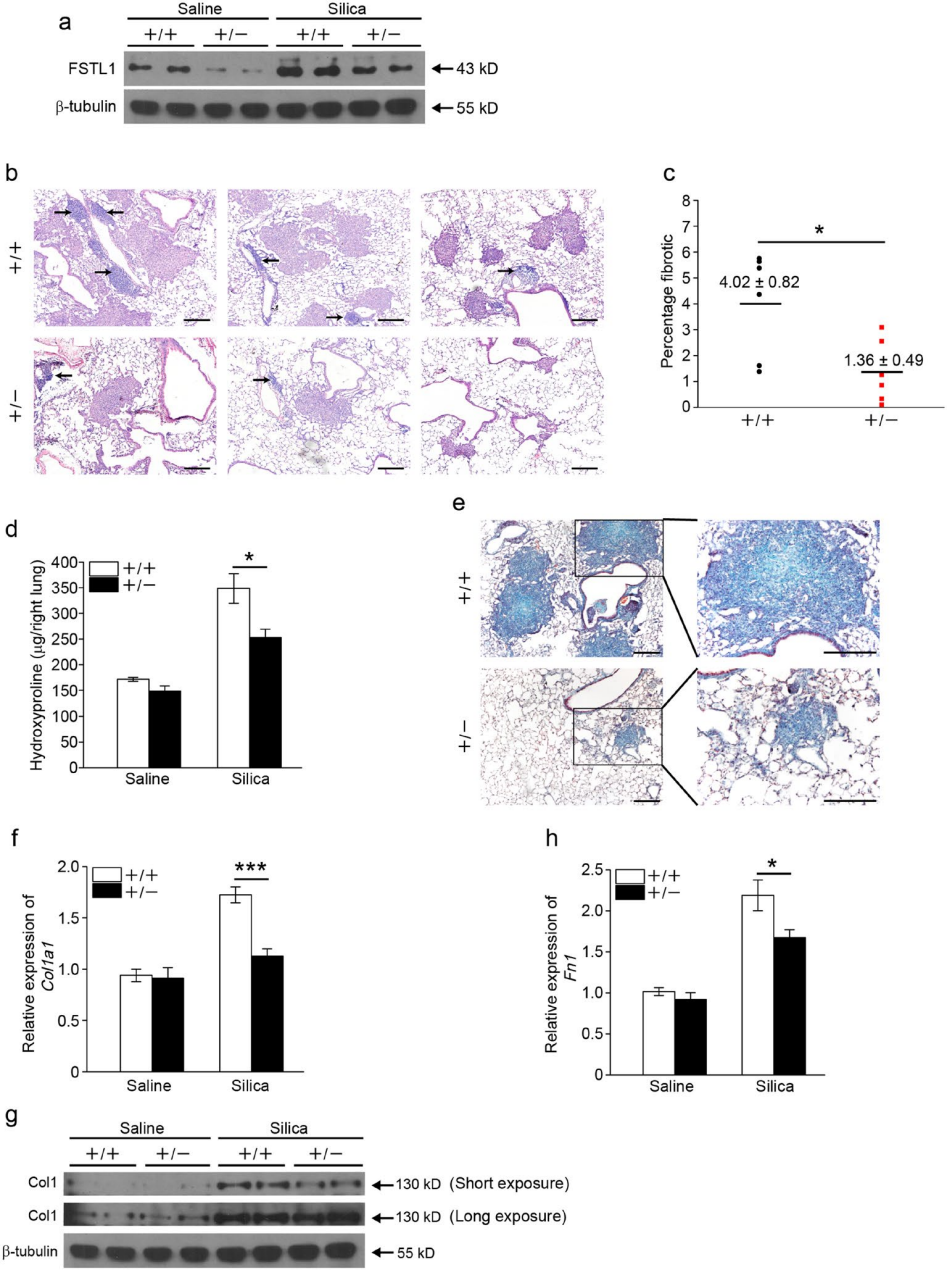
*Fstl1* ^+/−^ mice have an attenuated pulmonary fibrosis phenotype. (**a**) Western blot analysis of FSTL1 expression in lung tissues of *Fstl1* ^+/−^ and WT mice 21 days after saline or silica injury. β-tubulin was used as a loading control. (**b**) H&E staining of lung sections of *Fstl1* ^+/−^ and WT mice 21 days after silica injury. Representative images of the staining are shown. Arrows show inflammatory cells. (n = 6 per group; scale bars, 200 μm). (**c**) Lung fibrotic score analysis of the lung sections from *Fstl1* ^+/−^ and WT mice 21 days after silica injury (n = 6 per group; *P < 0.05 by one-way ANOVA followed by Student’s *t* test). The fibrotic area is presented as a percentage. (**d**) Hydroxyproline contents in lung tissues from *Fstl1* ^+/−^ and WT mice were measured 21 days after saline or silica injury (n = 6 per group; *P < 0.05 by one-way ANOVA followed by Student’s *t* test). (**e**) Masson trichrome staining of lung sections of *Fstl1* ^+/−^ and WT mice 21 days after silica treatment. Representative images of the staining are shown (n = 6 per group; scale bars, 200 μm). (**f**) qRT-PCR analysis of *Col1a1* mRNA expression in lung tissues from *Fstl1* ^+/−^ and WT mice 21 days after saline or silica injury (n = 3 per group; ***P < 0.001 by one-way ANOVA followed by Student’s *t* test). (**g**) Western blot analysis of type I collagen (Col1) expression in lung tissues from *Fstl1* ^+/−^ and WT mice 21 days after saline or silica injury (n = 3 per group). β-tubulin was used as a loading control. (**h**) qRT-PCR analysis of *fibronectin* (*Fn1*) mRNA expression in lung tissues from *Fstl1* ^+/−^ and WT mice 21 days after saline or silica injury (n = 3 per group; *P < 0.05 by one-way ANOVA followed by Student’s *t* test).

Silica is a well-established agent for inducing pulmonary inflammation and fibrosis^25^. To better understand the biological significance of the inducible expression of Fstl1 in the fibrotic process, we examined the inflammatory and fibrotic responses to silica-induced lung injury in *Fstl1*-dificient mice. Because homozygous *Fstl1* ^−/−^ mice die of respiratory failure shortly after birth^21^, *Fstl1* haplodeficient (*Fstl1* ^+/−^) mice were used in this study. The FSTL1 protein was significantly less in lung tissues of *Fstl1* ^+/−^ mice, and the increased FSTL1 protein after silica treatment was reduced in *Fstl1* ^+/−^ mice (Fig. 2a). Marked development of inflammation and subsequent formation of well-defined fibrotic nodules were observed in WT lungs after treatment with silica for 21 days, whereas, the fibrotic nodules in *Fstl1* ^+/−^ mice were much smaller and less frequent (Fig. 2b). Quantification of fibrotic lung sections by a blinded pathologist illustrated the attenuated fibrosis in *Fstl1* ^+/−^ mice when compared with littermate WT controls (Fig. 2c). These observations suggest that *Fstl1* deficiency can protect mice from silica-induced lung injury, and *Fstl1* ^+/−^ mice exhibit reduced lung fibrosis. The attenuated lung fibrosis in *Fstl1* ^+/−^ mice was further demonstrated by decreased collagen accumulation, as determined by hydroxyproline content (Fig. 2d) and Masson’s trichrome staining (Fig. 2e). Furthermore, qRT-PCR and western blot analyses revealed the decreased mRNA and protein expressions for type I collagen (Col1) and fibronectin (Fn1) in lung tissues from silica-injured *Fstl1* ^+/−^ mice than those of littermate WT controls (Fig. 2f–h). Our data support the role of Fstl1 in promoting fibrogenesis after silica-induced lung injury.

### *Fstl1*^+/−^ mice exhibit attenuated inflammation after silica injury

**Figure 3:**
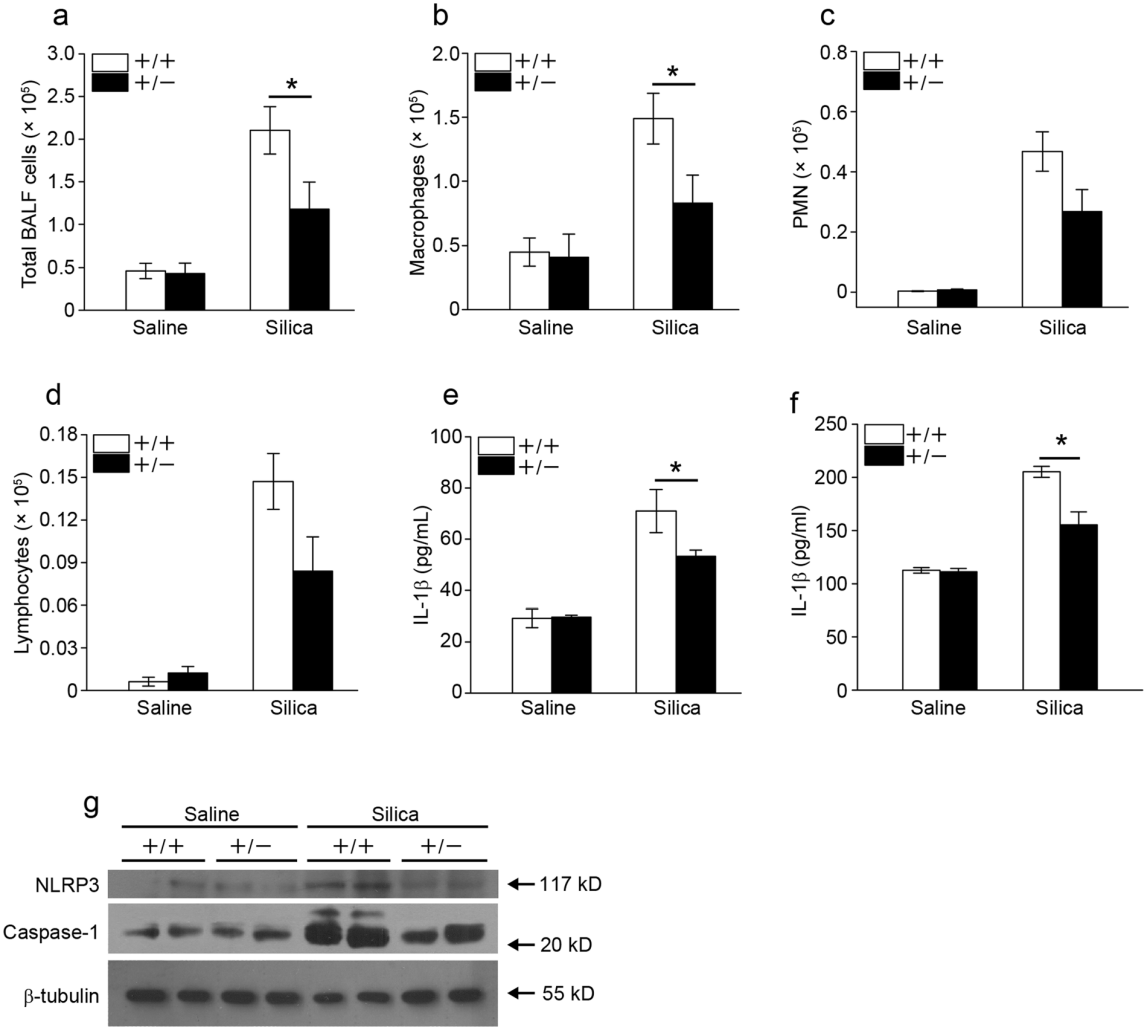
*Fstl1* ^+/−^ mice have an attenuated pulmonary inflammatory response. (**a–e**) *Fstl1* ^+/−^ and their WT littermate mice were intratracheally exposured to saline or 200 mg/Kg silica, bronchoalveolar lavage fluid (BALF) were collected from *Fstl1* ^+/−^ and WT mice 7 days after administration of saline or silica. (**a**) The number of total BALF cells was determined by hemocytometer (n = 7 per group; *P < 0.05 by one-way ANOVA followed by Student’s *t* test). (**b–d**) The differential cell counts in BALF were determined according to standard morphologic criteria. (**b**) Macrophages (n = 7 per group; *P < 0.05 by one-way ANOVA followed by Student’s *t* test). (**c**) PMNs (Polymorphonuclear neutrophils, n = 7 per group). (**d**) Lymphocytes (n = 7 per group). (**e**) The level of cytokine IL-1β in BALF was detected by ELISA assay (n = 7 per group; *P < 0.05 by one-way ANOVA followed by Student’s *t* test). (**f–g**) *Fstl1* ^+/−^ and their WT littermate mice were intratracheally exposured to saline or silica, lung tissues were collected from *Fstl1* ^+/−^ and WT mice 7 days after administration of saline or silica. (**f**) The level of cytokine IL-1β in lung tissues was detected by ELISA assay (n = 4 per group; *P < 0.05 by one-way ANOVA followed by Student’s *t* test). (**g**) The levels of NLRP3 and caspase-1 (p20) in lung tissues were determined by western blot analysis. β-tubulin was used as a loading control.

Exposure to silica initially causes lung inflammation, which is characterized by infiltration of inflammatory cells and subsequently release of various cytokines. Previous studies have implicated a role of Fstl1 in inflammatory response in rheumatoid arthritis^17^ and in heart allograft rejection^19^. We investigated the role of Fstl1 in the inflammatory response to silica-induced lung injury. We observed an increase in total inflammatory cells after silica treatment in the bronchoalveolar lavage fluid (BALF) from both WT and *Fstl1* ^+/−^ lungs over 7 days after silica treatment. However, a significant reduction in total inflammatory cells was observed in *Fstl1* ^+/−^ mice as compared with WT controls (Fig. 3a). Thus, the attenuated lung fibrosis developed in *Fstl1* ^+/−^ mice was associated with a significant decrease in inflammatory cell recruitment. This observation led us to further examine the recruitment of specific subsets of inflammatory cells following silica-induced lung injury. Differential counts of BALF cells demonstrated fewer macrophages in *Fstl1* ^+/−^ mice after silica injury (Fig. 3b). Although the reduced recruitment of neutrophils and lymphocytes was observed in *Fstl1* ^+/−^ mice, the differences between *Fstl1* ^+/−^ mice and WT controls were modest (Fig. 3c and d). We next measured the cytokine IL-1β, a powerful mediator of sterile inflammatory response^26^, whose dysregulation has been reported in lung tissue of silicosis patients^27^ and in silica model of mice^28^. As expected, IL-1β levels were lower in BALF of *Fstl1* ^+/−^ mice (Fig. 3e), as well as in lung tissues (Fig. 3f), at day 7 after silica treatment when compared to their WT controls. These data suggest that Fstl1 promotes silica-induced lung fibrosis, partially by regulating inflammation in response to tissue injury.

IL-1β is synthesized mainly by monocytes/macrophages, as an inactive precursor form^29^. Its biological activity is directly dependent on the cleavage by caspase-1 in NLRP3 inflammasome^30–32^. To further determine whether Fstl1 can regulate the activation of NLRP3 inflammasome *in vivo*, we examined the expression of the NLRP3 inflammasome components at protein level. Indeed, we observed the increased activation of NLRP3 inflammasome in response to silica stimulation, as indicated by increased levels of NLRP3 and Caspase-1 in lung tissues (Fig. 3g). However, the increased levels of NLRP3 and Caspase-1 were reduced in *Fstl1* ^+/−^ mice when compared with their WT littermates (Fig. 3g). These data collectively indicate that Fstl1 promotes silica-induced lung inflammation by positively regulating the activation of NLRP3 inflammasome and subsequently production of IL-1β in macrophages.

### *Fstl1*^+/−^ mice have less myofibroblasts accumulation after silica exposure

**Figure 4:**
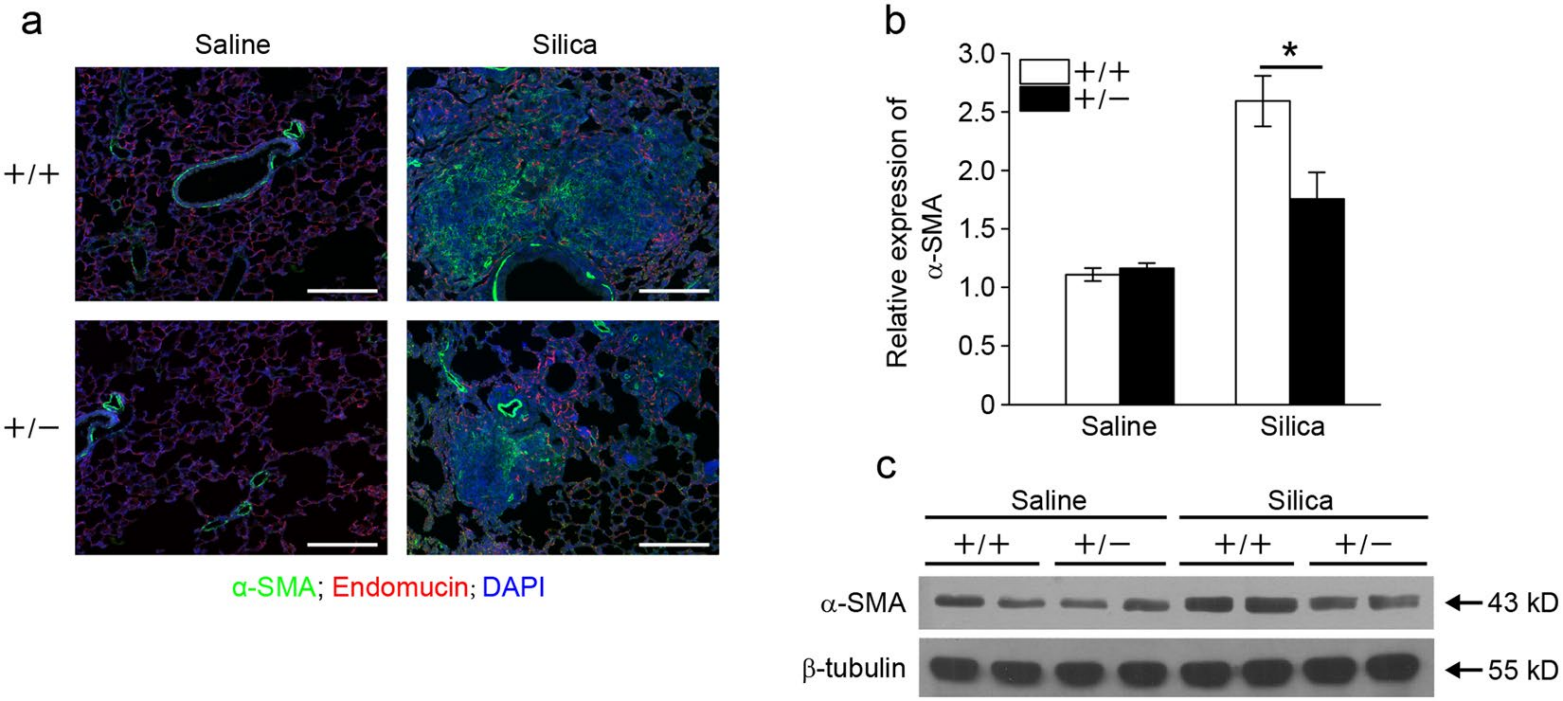
*Fstl1* ^+/−^ mice have less myofibroblast accumulation after silica exposure. (**a**) Immunofluorescence analysis of α-SMA expression in lung sections of *Fstl1* ^+/−^ and WT mice 21 days after saline or silica exposure. Representative images of the staining are shown. (α-SMA, green; Endomucin, red; nucleus, blue; scale bars, 200 μm). (**b**) qRT-PCR analysis of *α-SMA* mRNA expression in lung tissues from *Fstl1* ^+/−^ and WT mice 21 days after saline or silica treatment (n = 3 per group; *P < 0.05 by one-way ANOVA followed by Student’s *t* test). (**c**) Western blot analysis of α-SMA expression in lung tissues from *Fstl1* ^+/−^ and WT mice 21 days after saline or silica treatment. β-tubulin was used as a loading control.

To examine whether the attenuated fibrotic phenotype in silica-treated *Fstl1* ^+/−^ mice is associated with the accumulation of myofibroblasts, we stained lung sections with anti-α-SMA antibody, a marker for newly appearing myofibroblasts. *Fstl1* ^+/−^ mice showed a remarkable decrease in cells immunofluorescence staining positive for α-SMA after 21 days silica treatment (Fig. 4a). Consistently, qRT-PCR (Fig. 4b) and western blot (Fig. 4c) analyses revealed the decreased mRNA and protein expression of α-SMA in lung tissues of *Fstl1* ^+/−^ mice when compared with those of WT littermates. Taken together, our data suggest that Fstl1 plays a role in promoting fibrogenesis after silica-induced lung injury, possibly by facilitating fibroblast/myofibroblast accumulation to areas of tissue injury.

### Fstl1 modulates myofibroblast differentiation via facilitating TGF-β1 signaling

**Figure 5:**
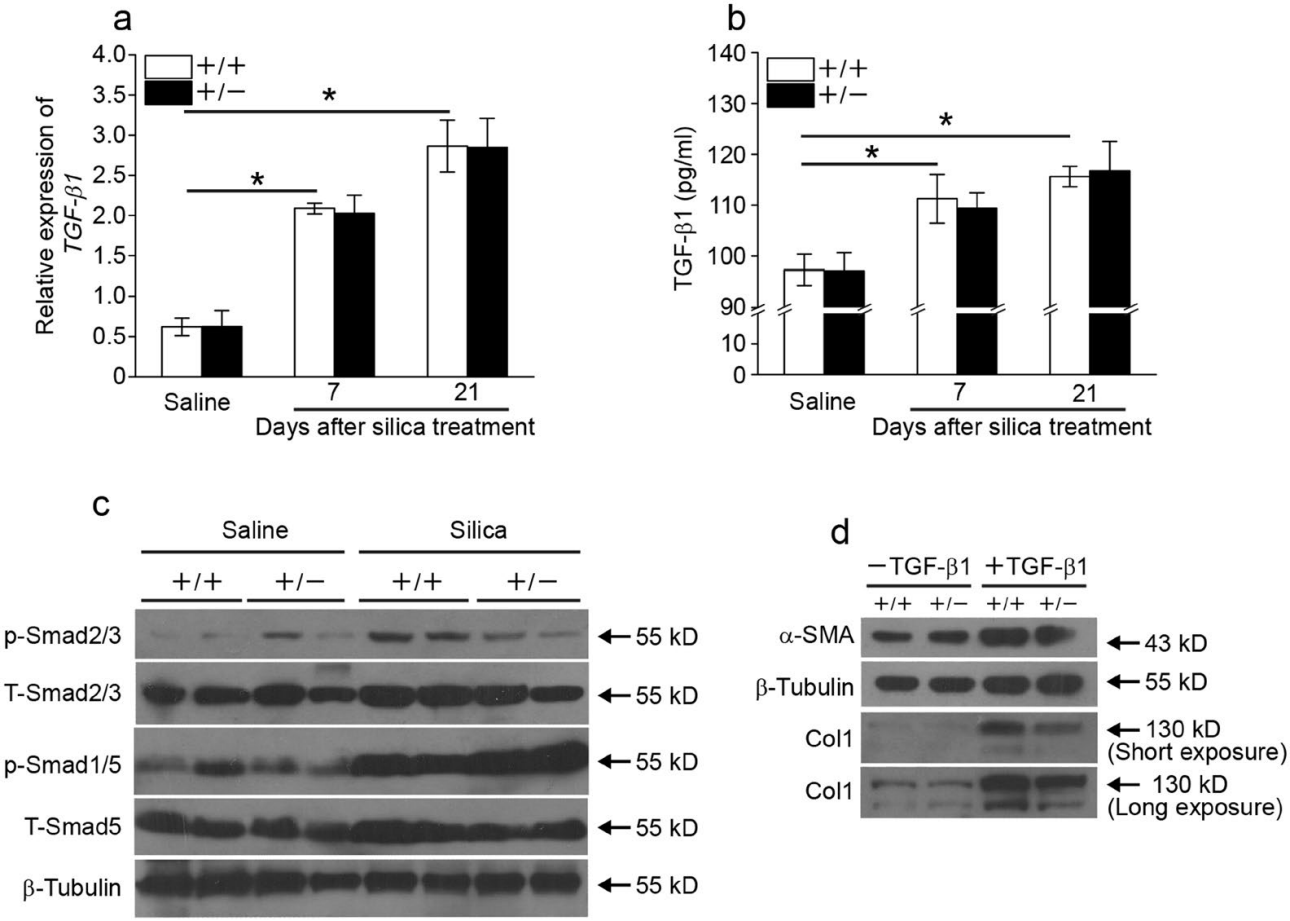
Fstl1 modulates myofibroblast differentiation via facilitating TGF-β1 signaling. (**a**) qRT-PCR analysis of TGF-β1 mRNA expression in lung tissues of *Fstl1* ^+/−^ and WT mice at indicated time after saline or silica exposure (n = 4 per group; *P < 0.05 by one-way ANOVA followed by Student’s *t* test). (**b**) ELISA analysis of active form of TGF-β1 protein in lung tissues of *Fstl1* ^+/−^ and WT mice at indicated time after saline or silica exposure (n = 4 per group; *P < 0.05 by one-way ANOVA followed by Student’s *t* test). (**c**) The levels of phosphorylation of Smad2/3 (p-Smad2/3), total Smad2/3 (T-Smad2/3), phosphorylation of Smad1/5 (p-Smad1/5) and total Smad1/5 (T-Smad1/5) in lung tissues of of *Fstl1* ^+/−^ and WT mice 21 days after saline or silica exposure were determined by western blot analysis. β-tubulin was used as a loading control. (**d**) Primary lung fibroblasts from *Fstl1* ^+/−^ and WT mice were treated with 5 ng/ml TGF-β1. Protein expressions of α-SMA in cell extracts and type I collagen (Col1) in medium 24 h after TGF-β1 treatment were determined by western blot analysis. β-tubulin was used as a loading control.

TGF-β1, as well as its signaling, plays a central role in the initiation and progression of different types of fibrosis, including silicosis. To define the mechanisms whereby *Fstl1* deficiency results in the attenuated myofibroblasts and subsequent fibrogenesis, we examined the regulation of Fstl1 on TGF-β1 expression and Smad2/3-mediated TGF-β1 signaling. WT and *Fstl1* ^+/−^ mice displayed comparable levels of mRNA and active protein form of TGF-β1 (Fig. 5a and b) by 21 days after silica treatment, however, western blotting revealed attenuated phosphorylation levels of Smad2/3 in lung tissues *Fstl1* ^+/−^ mice (Fig. 5c), indicating that Fstl1 promoted TGF-β1/Smad2/3 signaling *in vivo* but not their expression and activation. We then isolated primary lung fibroblasts from WT and *Fstl1* ^+/−^ lungs, and used an *in vitro* model TGF-β1-induced myofibroblast differentiation to define the role of Fstl1. As detected by western blotting, TGF-β1-induced myofibroblast differentiation was demonstrated by the increased α-SMA protein levels and subsequent type I collagen (Col1) production in WT lung fibroblasts (Fig. 5d). This induced myofibroblast differentiation was greatly abolished in *Fstl1* ^+/−^ lung fibroblasts (Fig. 5d). A TGF-β1/BMP signaling imbalance has been suggested to play a role in fibrogenesis. We measured Smad1/5/8-mediated BMP4 signaling in silica-injured lung tissues. Compared to the decreased phosphorylation levels of Smad2/3, western blotting showed higher phosphorylation levels of Smad1/5 in *Fstl1* ^+/−^ lungs (Fig. 5c). This is also in agreement with our recent study using a bleomycin mouse model^24^ and suggests that the restored TGF-β1/BMP balance may be the molecular underpinnings for the attenuated fibrogenesis in *Fstl1* ^+/−^ mice.

### Blockage of FSTL1 with a neutralizing antibody attenuates silica-induced lung inflammation and fibrosis *in vivo*

**Figure 6:**
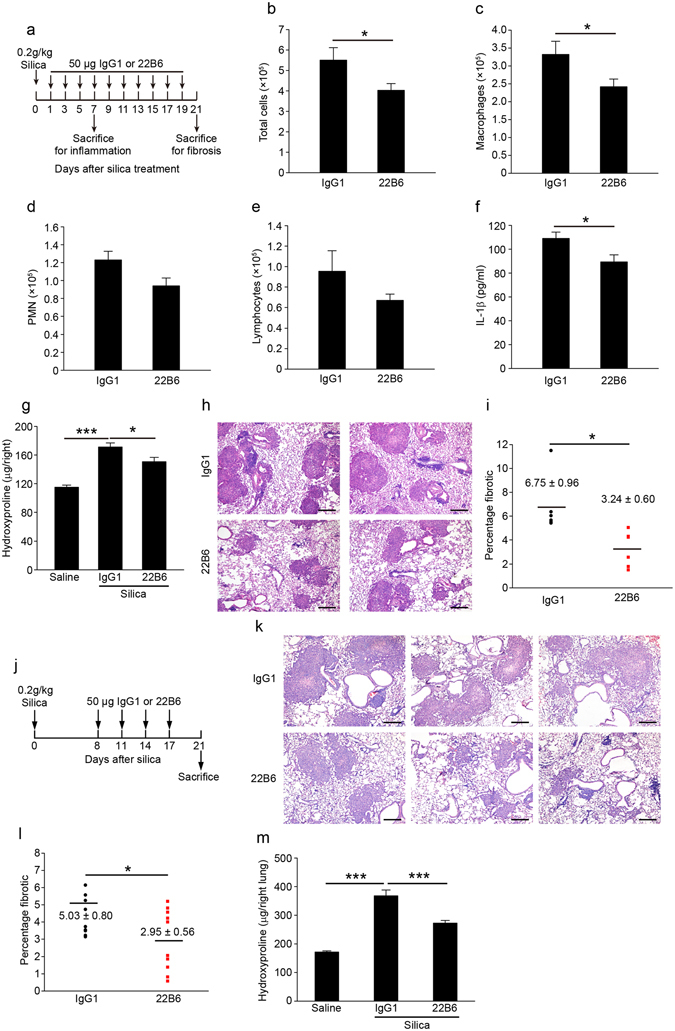
FSTL1-neutralizing antibody attenuates silica-induced lung inflammation and subsequent pulmonary fibrosis in mice. (**a**) In a FSTL1 blockage experiment, C57BL/6 mice were intraperitoneally injected with 22B6 mAb or IgG1 (n = 6 per group) every other day from 1 day after silica challenge till the mice were sacrificed on day 7 for inflammation analysis or day 21 for fibrosis analysis. (**b–f**) For inflammation analysis, (**b**) the number of total BALF cells was determined by hemocytometer (*P < 0.05 by one-way ANOVA followed by Student’s *t* test). (**c–e**) The differential cell counts in BALF were determined according to standard morphologic criteria. (**c**) Macrophages (*P < 0.05 by one-way ANOVA followed by Student’s *t* test). (**d**) PMNs. (**e**) Lymphocytes. (**f**) The level of cytokine IL-1β was detected by ELISA assay (*P < 0.05 by one-way ANOVA followed by Student’s *t* test). (**g**–**i**) For fibrosis analysis, (**g**) hydroxyproline contents in lung tissues were measured (*P < 0.05, ***P < 0.001 by one-way ANOVA followed by Student’s *t* test). (**h**) Representative images of the H&E staining of lung sections are shown (Scale bars, 200 μm). (**i**) Lung fibrotic score analysis of the lung sections. The fibrotic area is presented as a percentage (*P < 0.05 by one-way ANOVA followed by Student’s *t* test). (**j**) Interventional dosing regimen of lung fibrosis model. C57BL/6J mice were intraperitoneally injected with 22B6 mAb or IgG1 (n = 10 per group) at indicated time after silica exposure, and lungs were harvested on day 21. (**k**) Representative images of the H&E staining of lung sections are shown (Scale bars, 200 μm). (**l**) Lung fibrotic score analysis of the lung sections. The fibrotic area is presented as a percentage (*P < 0.05 by one-way ANOVA followed by Student’s *t* test). (**m**) Hydroxyproline contents in lung tissues (***P < 0.001 by one-way ANOVA followed by Student’s *t* test).

To explore the role of increased Fstl1 in silica-induced lung fibrosis, C57BL/6J mice were intraperitoneally injected with FSTL1 neutralizing antibody (22B6 mAb)^24^ every other day from day 1 to 19 after silica treatment (Fig. 6a). The mice were sacrificed at day 7 for inflammation analysis, and at day 21 for fibrosis analysis, respectively. Lung inflammation was significantly attenuated in mice treated with 22B6 mAb, when compared with that with isotype-matched control antibody, as indicated by reduced total inflammatory cells, macrophages and pro-inflammatory cytokine IL-1β in BALF (Fig. 6b–f). Similarly, the mice treated with 22B6 mAb showed an attenuated lung fibrosis. Collagen accumulation was significantly reduced in mice treated with 22B6 mAb, compared with those treated with isotype-matched control antibody (Fig. 6g). Quantification of fibrotic lung sections by a blinded pathologist illustrated a similar result (Fig. 6h and i). These data suggest that blockage of FSTL1 with a neutralizing antibody attenuates silica-induced lung inflammation and subsequent fibrosis *in vivo*.

As *Fstl1* deletion on myofibroblasts also had antifibrotic effects in the lung (Fig. 5d), we assessed whether 22B6 mAb administration at day 8, 11, 14, and 17 after intratracheal silica could prevent further progression of lung fibrosis (Fig. 6j). As expected, lung fibrosis was markedly decreased in mice treated with 22B6 Ab, as indicated by less fibrotic area (Fig. 6k and l) and lower collagen accumulation measured by hydroxyproline content (Fig. 6m). These data further support that endogenous production of FSTL1 following silica-induced lung injury regulates inflammatory and fibrogenic response.

## Discussion

Silicosis is a progressive pulmonary fibrosis disease caused by long-term inhalation of free crystalline silica. It is the main form of the occupational diseases worldwide with a median survival of 6 years from diagnosis^33^. Despite extensive research, the molecular mechanisms underlying remain poorly understood. Currently, few effective therapies exists^34^. The findings reported in this study add new insights into our understanding of the regulation of silicosis and demonstrate the pro-fibrotic role of Fstl1 using a silica mouse model. We provide data at clinical and animal levels, as well as proof of principle intervention to support a role of Fstl1 as a potential therapeutic target for silicosis.

We offered the following lines of evidence to demonstrate the pro-fibrotic role of Fstl1 in silicosis. Circulating level of FSTL1 was higher in serum of patients with silicosis. Fstl1 was induced in mouse lung and accumulated in fibrotic nodules in response to silica injury. Targeting deletion of *Fstl1* significantly attenuated inflammation and lung fibrosis in mouse model of silicosis. Blockage of FSTL1 with a neutralizing antibody reduced silica-induced lung inflammation and subsequent fibrosis in mice *in vivo*.

We found that Fstl1 regulated lung fibrosis of silicosis partially through fibroblast activation and differentiation. Myofibroblasts, with heightened capacity for contractility and production of ECM, represent an activated phenotype of fibroblasts^35^ that play a critical role in fibrogenesis of different types of pulmonary fibrotic diseases^36^. The differentiation of myofibroblasts is a complex, highly integrated process that mediated by various factors, such as TGF-β1, one of the key cytokines involved in pulmonary fibrosis. Recently, we have identified Fstl1 as a novel pro-fibrotic factor in idiopathic pulmonary fibrosis (IPF) and a mouse model of bleomycin injury^24^. Fstl1 promotes the accumulation and differentiation of myofibroblasts and subsequent fibrosis via positive regulation of TGF-β1 signaling. In this study, we showed the accumulation of myofibroblasts was significantly reduced in *Fstl1* ^+/−^ mice after silica injury, indicated by the decreased expression of α-SMA (a marker of myofibroblast). Accordingly, TGF-β1/Smad2/3 signaling, consistent to α-SMA expression, decreased in *Fstl1* ^+/−^ lungs after silica injury. This is in agreement with our recent study^24^ and suggests a common role of Fstl1 in the accumulation and differentiation of myofibroblasts via positive regulation of TGF-β1 signaling. Fstl1 may be a broad pro-fibrotic factor in different types of pulmonary fibrotic diseases and would serve as a common therapeutic target for ILDs.

Persistent inflammation often drives fibrotic progression, which is an important process of silicosis. The inhalant small particles deposit in distal alveoli, where they are ingested by alveolar macrophages, and initiate inflammation. Recent evidences suggest that silica particles are capable of activating the NLRP3 inflammasome, which promotes the production of mature IL-1β in macrophages. Hirsch, *et al.* ^37^ reported that Fstl1 is induced in response to endotoxin and enhances NLRP3 inflammasome-mediated IL-1β from macrophages. Here we demonstrated that increased Fstl1 in silica mouse model also enhances NLRP3 inflammasome-mediated IL-1β from macrophages. Haplodeletion of *Fstl1* in mice or blockage of FSTL1 with neutralizing antibody in mice reduced silica-induced NLRP3 expression, caspase-1 activity, and subsequent IL-1β secretion *in vivo*. Although ours and previous studies have implicated the role of Fstl1 in inflammatory response in silica model, LPS model^37, 38^, as well as in rheumatoid arthritis^17^ and heart allograft rejection^19^, we have reported that Fstl1 promotes bleomycin-induced lung fibrosis without significantly affecting the inflammatory response. Our data suggest that Fstl1 has different effects on fibrogenesis in different types of ILDs.

FSTL1 is an extracellular glycoprotein and its circulating levels are elevated in patients with heart failure and rheumatoid arthritis^18^, suggesting that FSTL1 can serve as a useful biomarker for cardiac disease^39^. In our experimental silicosis mouse model, we detected an increased circulating FSTL1 level in mouse serum after silica injury. Consistently, we also measured elevated levels of FSTL1 protein in serum of patients with silicosis, when compared with those of healthy individuals. However, whether FSTL1 may serve as a potential peripheral blood biomarker for silicosis still needs more intensively studies.

In summary, we have demonstrated that Fstl1 is an important pro-inflammatory and pro-fibrotic factor in silica induced lung injury and targeting deletion of *Fstl1* attenuated the pulmonary inflammation and fibrosis in response to lung injury. Additionally, we provide the *in vivo* animal evidences to support the therapeutic role of Fstl1 for lung fibrosis. We also showed the elevated circulating FSTL1 levels in serum of patients with silicosis. The correlation between circulating FSTL1 levels and patients’ lung function is actively pursued in our laboratories. This study and our continuing efforts will provide novel insights into the understanding of pathology of silicosis and a novel therapeutic target and/or a diagnostic biomarker for management of the fibrotic process.

## Materials and Methods

### Ethics Statement

All the participants in this study signed informed consent before enrolled the study and this study obtained the authorization from the Ethics Committees of Beijing Chao-Yang Hospital of Capital Medical University. The investigations were approved by the Institutional Review Boards of Beijing Chao-Yang Hospital of Capital Medical University and conducted in accordance with the ethical standards of Beijing Chao-Yang Hospital of Capital Medical University and the World Medical Association Declaration of Helsinki. All experimental methods involving human subjects were completed in accordance with the relevant guidelines and regulations. All mice were housed and bred in a specific pathogen-free environment at Nankai University and treated in strict accordance with protocols approved by the Animal Care and Use Committee at Nankai University (Approval Number: 20140008). All efforts were made to minimize suffering.

### Subjects

Lung tissue sample from patient with silicosis obtained from lung biopsies, used for hematoxylin and eosin (H&E) staining and immunohistochemistry (IHC) analysis, was from Beijing Chao-yang hospital. This silicosis patient was diagnosed by physical examination, pulmonary function studies, chest high-resolution computed tomography (HRCT), and BAL findings according to the respective diagnostic criteria for this disease (Diagnostic criteria of pneumoconiosis, 2009, China). This silicosis patient is a 56-yr-old man. The FVC was 81.8% predicted and FEV1 was 66.4% predicted. The peripheral blood from patients with silicosis collected using a vacuum blood collection tube was from Beijing Chao-yang hospital. The blood samples were statically kept at 4 °C for 2 h, and then centrifuged at 3000 rpm for 10 min at 4 °C. The serum was transferred to a clean tube for further analysis. The clinical characteristics of patients and normal control individuals were shown in Table 1.

**Table 1:** Clinical characteristics of the study groups.

	Normal (n = 21)	Silicosis (n = 37)
Age, years	56.8 ± 1.1	57.7 ± 2.1
Gender		
Male, n(%)	11(52.4)	22(59.5)
Female, n(%)	10(47.6)	15(40.5)
Stage		
I, n(%)	—	13(35.1)
II, n(%)	—	8(21.6)
III, n(%)	—	16(43.3)
Serum FSTL1 (ng/mL)	52.49 ± 2.99	65.13 ± 4.28^*^

Values are mean ± SEM.

*P < 0.05, significant difference compared with normal.

### ELISA assay for human FSTL1 protein in serum

100 μL serum samples were added to the wells of the assay plate, which was coated with 5 μg/mL human FSTL1 capture antibody (R&D system, AF1694). The plate was incubated at 4 °C overnight, then 2.5 μg/mL biotinylated human FSTL1 detection antibody (R&D system, MAB1694) was added followed by washing with wash buffer and the plate was incubated for 1 hour at room temperature. Streptavidin-HRP was added to the wells of assay plate and incubated for another 30 min at room temperature. After washing the plate again, TMB substrate was added to each well and incubated in the darkness for 15 min at room temperature. The absorbance values of the wells at 450 nm and 570 nm were obtained after the stop solution was added. The ELISA assay was performed in duplicate for every sample.

### Mice

The *Fstl1* ^+/−^ mice were generated as previously described^21^, and have been back crossed onto the C57BL/6J genetic background for at least 12 generations before used. Healthy *Fstl1* ^*+/+*^ and *Fstl1* ^+/−^ mice at 8-week-old were used for the experiments. The 8-week-old wild type C57BL/6J mice were purchased from Vital River Laboratories (Beijing China).

### Silica preparation and exposure

Silica was purchased from US SILICA (MIN-U-SIL 5, Mill Creek, OK, USA). The content of the SiO_2_ dust was >99.2%, and the particle median diameter was 1.4 μm. The silica dust was maintained in an oven at 180 °C for 2 h to remove endotoxin and water, then weighed and suspended in phosphate-buffered saline (PBS) to make the final concentration at 200 mg/mL. Suspensions were sonicated for 30 min and vortexed for 5 seconds before used. After anaesthetized with intraperitoneal injection of 7.5% chloral hydrate (Sangon, Shanghai, China), the trachea of mice was exposed by opening the neck skin and blunt dissection. Mice received the suspension at 200 mg/Kg body weight by intratracheal administration using a 22G needle (Optiva, USA). The site of surgery was sutured and cleaned with ethanol. Mouse lungs were harvested at the different time-points.

### FSTL1-neutralizing antibody treatment *in vivo*

The experiment of FSTL1-neutralizing antibody treatment *in vivo* was performed as previously described^24^. In brief, the 8-week-old C57BL/6J mice were intratracheally administrated with 200 mg/Kg silica. FSTL1-neutralizing antibody (clone 22B6) or its control isotype antibody (IgG1) was intraperitoneally injected (50 μg/mouse/each time) at indicated time after silica treatment. For inflammation analysis, the mouse lungs were harvested on day 7 after silica injury, and for fibrosis analysis, the mouse lungs were harvested on day 21 after silica injury. Left lung was sectioned for H&E staining to assess the degree of fibrosis and right lungs were used for measuring collagen contents with a conventional hydroxyproline method.

### Mouse blood collection from orbital sinus

Mouse blood samples were collected as previously described^40^. Briefly, a microhematocrit tube was introduced to the canthus of the anesthetized mice. The blood flowed into the tube when the microhematocrit tube was slightly advanced and rotated. The blood samples were statically kept at room temperature for 1 h, and then centrifuged at 3000 rpm for 10 min at 4 °C. The serum was transferred to a clean tube and stored at −80 °C for further analysis.

### Bronchoalveolar lavage fluid (BALF) collection and differential cell counts

BAL fluid was collected as previously described^24^. Briefly, the trachea was cannulated and lavaged three times with 0.8 mL cold sterile PBS. The BALF aliquots were transferred into three sterilized tubes, followed by centrifugation at 1000 rpm for 10 min at 4 °C. The supernatant of the first tube was separated for later analysis. The pellets of all tubes were washed with PBS and then resuspended in 200 μL PBS for total cells and differential cell counting. A hemocytometer was used to quantify the total cells. For the differential cell counts, smears of each suspension were stained with H&E staining, and 500 cells were categorized as macrophages, neutrophils or lymphocytes using standard morphologic criteria.

### ELISA

The levels of mouse cytokine IL-1β (Biolegend, USA) in BALF, TGF-β1 (USCN, China) in whole lung tissue lysates and FSTL1 (USCN, China) proteins in serum were measured with commercial ELISA kit according to the manufacturer’s instructions. Briefly, 100 μL BALF samples were added to the wells of the assay plate coated with capture antibody. After overnight incubation, detection antibody was added to the assay plate, and then streptavidin-HRP was added to the wells. The ELISA assay was developed with TMB substrate.

### AM, AEC and fibroblast isolation and culture

Alveolar macrophages (AMs) were isolated from saline- and silica- treated mice as previously described^41^. Briefly, lungs were lavaged three times with 1 ml sterile PBS and the lavage fluid was collected in a 15 ml conical tube. The BALF was centrifugated at 1000 rpm for 10 min at 4 °C. The supernatant was removed and the pellet was resuspended in 1640 medium supplement with 10% FBS and antibiotics. Cells were cultured in 5% CO_2_ at 37 °C in a humidified atmosphere. After 24 h, the adherent cells were collected for further analysis. Primary alveolar epithelial cells (AECs) and fibroblasts were isolated from saline- and silica- treated mice and cultured in DMEM supplement with 10% FBS and antibiotics in 5% CO_2_ at 37 °C in a humidified atmosphere as previously described^24^. After 3–5 days cultured, primary AECs and fibroblasts were used for further analysis.

### Hydroxyproline assay

The conventional hydroxyproline method was used for measuring collagen contents in lungs. The right lungs were dried at 120 °C overnight and then acid hydrolyzed in sealed, oxygen-purged glass ampules containing 3 mL HCl at 120 °C for 16 h. Samples were filtered through a 5.0 μm syringe-driven filter, and then the pH value was adjusted to 6.5–8.0 by using NaOH. The hydroxyproline analysis was performed using chloramine-T spectrophotometric absorbance as previously described^42^.

### Histology, immunohistochemistry and immunofluorescence

The left lungs were perfused with 10% neutral buffered formalin through a tracheal cannula. The tissues were fixed overnight, and then dehydrated, embedded in paraffin. 5 μm-thick sections were mounted on slides, followed by deparaffinization. The sections were stained with H&E for quantification of pulmonary fibrosis or Masson’s trichrome to visualize collagen as described previously^24^. Immunohistochemical (IHC) analyses for mouse and human FSTL1 (R&D System) were performed according to a previous paper. In brief, the sections were deparaffinization and rehydrated. After the antigen was recovered by high-pressure heating with 10 mM citrate buffer (pH 6.0) and treated with 3% H_2_O_2_ in PBS for 10 min and blocked with 5% normal goat serum, the tissue sections were incubated with primary antibody at 4 °C overnight. After extensive wash, the sections were incubated with HRP-polymer secondary antigens for 10 min at room temperature and then developed with DAB solution. For immunofluorescence analysis, the sections were incubated with a-SMA (sigma) primary antibody at 4 °C overnight, and then the Alexa Fluor-488-conjugated and Alexa Fluor-594-conjugated secondary antibodies (Jackson ImmunoResearch, USA) were used for immunofluorescent visualization. The nucleus was stained with DAPI (Santa Cruz Biotechnology), and then photographed with Zeiss Axio Imager Z1 microscope and analyzed by Zeiss software.

### Western Blotting

Western Blot analysis was performed following standard protocols as previously described^43^. Lung tissues were homogenized in 62.5 mM Tris Buffer, and then centrifuged at 12000 rpm for 10 min at 4 °C, and soluble supernatants were taken as whole tissue lysates. Equal amounts of protein mixed with sample buffer were separated on 10% SDS-PAGE gels, and the separated proteins were transferred onto a polyvinylidene fluoride (PVDF) transfer membrane at a constant voltage of 100 V for 100 min in ice-water mixture. The membranes were blocked with fat-free milk at room temperature for 1 h and incubated at 4 °C overnight with primary antibodies. The primary antibodies used in this study were anti-mouse FSTL1 (R&D system), anti-type I collagen (Abcam), α-SMA (sigma), NLRP3 and caspase-1 (Santa Cruz), β-tubulin (proteintech). After the HRP-conjugated secondary antibodies were incubated, protein signals were detected using the enhanced chemiluminescence (ECL) kit (Pierce Biotechnology, USA). All experiments were repeated in triplicate.

### RNA extraction and quantitative RT-PCR analysis

The total RNA was isolated from lung tissues using TRIzol Reagent according to the manufacturer’s instruction. Quantitative real-time reverse transcription-polymerase chain reaction (qRT-PCR) was performed as described previously^24^. Briefly, the first-strand cDNA was synthesized using random mixture primers and M-MLV reverse transcriptase. Real-time qPCR reactions were carried in a final volume of 20 μL containing SYBR Green PCR master mix, 10 ng cDNA, and 5 pmol primers. Gene expressions were determined relative to the endogenous reference gene (mouse *β-actin*) using the 2^−Δ(ΔCT)^ method. The sequences of qRT-PCR primers are described below: mouse *Fstl1* (NM_008047.5), 5′-TTATGATGGGCACTGCAAAGAA-3′ and 5′-ACTGCCTTTAGAGAACCAGCC-3′; *Fn1* (NM_010233.1), 5′-GTGTAGCACAACTTCCAATTACGAA-3′ and 5′-GGAATTTCCGCCTCGAGTCT-3′; *Col1a1* (NM_00774 2.3), 5′-CCAAGAAGACATCCCTGAAGTCA-3′ and 5′-TGCACGTCATCGCACAC A-3′; *a-SMA* (NM_007392.2), 5′-GCTGGTGATGATGCTCCCA-3′ and 5′-GCCCATTCCAACCATTACTCC-3′; *TGF-β1* (NM_011577.2), 5′-GAGCCCGAAGCGGACTACTA-3′ and 5′-TGGTTTTCTCATAGATGGCGTTG-3′; *β-actin* (NM_007393.3), 5′-AGGCCAACCGTGAAAAGATG-3′ and 5′-AGAGCATAGCCCTCGTAGATG G-3′.

### Statistical analysis

In this study, the SPSS software was used to evaluate statistical significance. Differences in measured variables between experimental and control group were assessed by using Student’s *t* test or Wilcoxon rank-sum test with nonparametric data. One-way ANOVA with Bonferroni test was used for multiple comparisons. All data are expressed as the means ± SEM. Results were considered statistically significant at *P* < 0.05.
