# Author Correction: Follistatin like-1 aggravates silica-induced mouse lung injury

**DOI:** 10.1038/s41598-024-59925-4

**Published:** 2024-04-26

**Authors:** Yinshan Fang, Si Zhang, Xiaohe Li, Fangxin Jiang, Qiao Ye, Wen Ning

**Affiliations:** 1 State Key Laboratory of Medicinal Chemical Biology, College of Life Sciences, Nankai University, Tianjin, China; 2 Department of Occupational Diseases and Toxicology, Beijing Chao-Yang Hospital, Capital Medical University, Beijing, China

Correction to: *Scientific Reports* 10.1038/s41598-017-00478-0, published online 24 March 2017

This Article contains an error in Figure 4, panel a, where the image from the “Saline” panel of “+/- group” is a shifted field of view from the “+/+” group. The correct Figure 4 and accompanying legend appear below.

**Figure 4:**
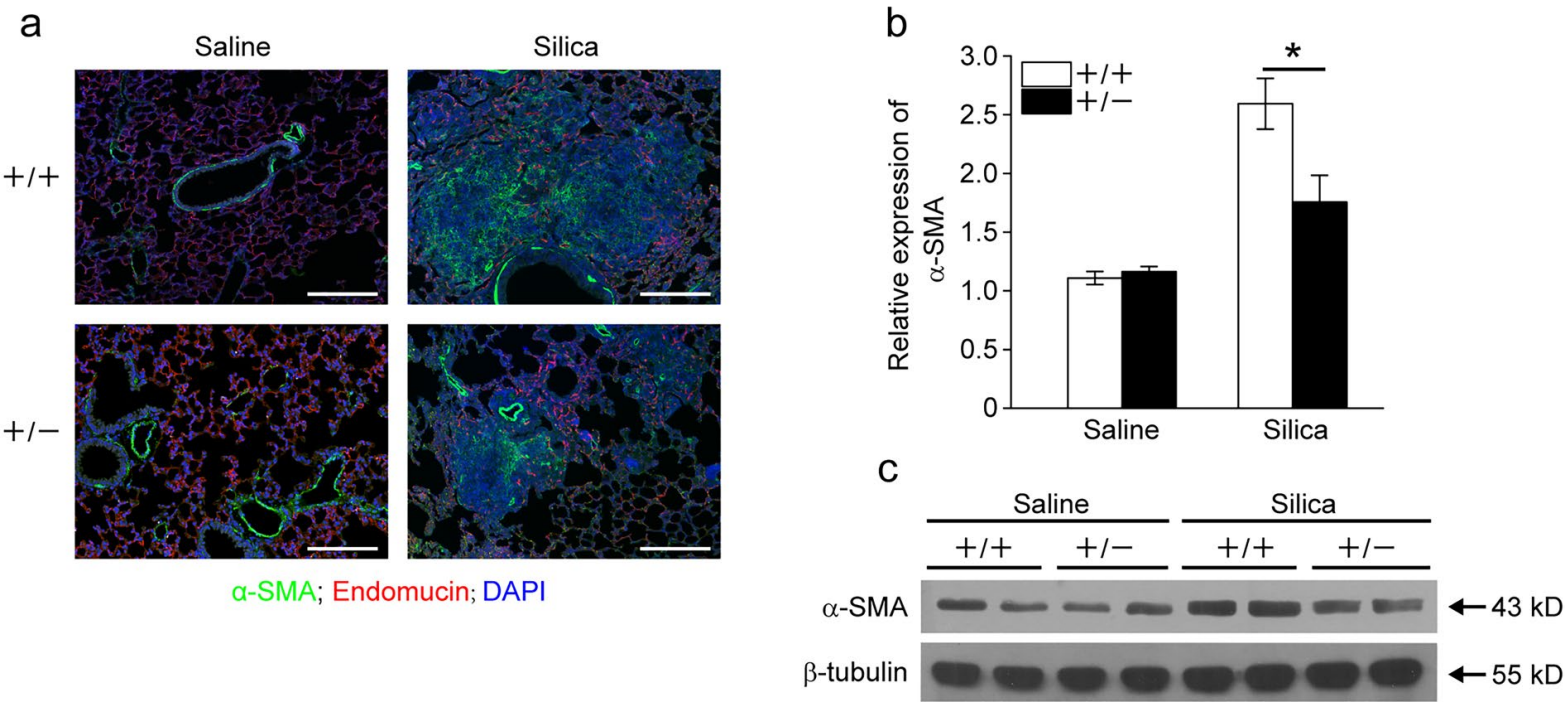
*Fstl1* ^+/−^ mice have less myofibroblast accumulation after silica exposure. (**a**) Immunofluorescence analysis of α-SMA expression in lung sections of *Fstl1* ^+/−^ and WT mice 21 days after saline or silica exposure. Representative images of the staining are shown. (α-SMA, green; Endomucin, red; nucleus, blue; scale bars, 200 μm). (**b**) qRT-PCR analysis of *α-SMA* mRNA expression in lung tissues from *Fstl1* ^+/−^ and WT mice 21 days after saline or silica treatment (n = 3 per group; *P < 0.05 by one-way ANOVA followed by Student’s *t* test). (**c**) Western blot analysis of α-SMA expression in lung tissues from *Fstl1* ^+/−^ and WT mice 21 days after saline or silica treatment. β-tubulin was used as a loading control.

